# Using Regularization to Infer Cell Line Specificity in Logical Network Models of Signaling Pathways

**DOI:** 10.3389/fphys.2018.00550

**Published:** 2018-05-22

**Authors:** Sébastien De Landtsheer, Philippe Lucarelli, Thomas Sauter

**Affiliations:** Systems Biology Group, Life Sciences Research Unit, University of Luxembourg, Belvaux, Luxembourg

**Keywords:** regularization, sparsity, clustering, network model, logical model, optimization

## Abstract

Understanding the functional properties of cells of different origins is a long-standing challenge of personalized medicine. Especially in cancer, the high heterogeneity observed in patients slows down the development of effective cures. The molecular differences between cell types or between healthy and diseased cellular states are usually determined by the wiring of regulatory networks. Understanding these molecular and cellular differences at the systems level would improve patient stratification and facilitate the design of rational intervention strategies. Models of cellular regulatory networks frequently make weak assumptions about the distribution of model parameters across cell types or patients. These assumptions are usually expressed in the form of regularization of the objective function of the optimization problem. We propose a new method of regularization for network models of signaling pathways based on the local density of the inferred parameter values within the parameter space. Our method reduces the complexity of models by creating groups of cell line-specific parameters which can then be optimized together. We demonstrate the use of our method by recovering the correct topology and inferring accurate values of the parameters of a small synthetic model. To show the value of our method in a realistic setting, we re-analyze a recently published phosphoproteomic dataset from a panel of 14 colon cancer cell lines. We conclude that our method efficiently reduces model complexity and helps recovering context-specific regulatory information.

## 1. Introduction

One goal of Systems Biology is to understand emerging functional properties of biological systems from the interactions of their components (Wolkenhauer, 2014). Such understanding would allow the design of new pharmacological strategies to treat diseases that arise when these systems do not function adequately, like cancer. One frequent approach is to map experimental measurements to the model variables of the system, and infer the most likely parametrization. To be useful, a well-parametrized model of a complex system should not only be able to predict non-obvious, non-linear behaviors, but also provide a mechanistic explanation for these behaviors and to suggest hypotheses about ways to control the system.

The most informative modeling approaches include prior information about the system (Aldridge et al., 2006). Classically, dynamical systems like regulatory networks of mammalian cells are modeled with systems of ordinary differential equations, describing in detail the status of chemical species like proteins or membrane receptors over time. Alternatively, logical models (Morris et al., 2010; Hill et al., 2012; Le Novère, 2015) were introduced several decades ago for the modeling of regulatory networks (Kauffman, 1969). As they are simpler in their formulation, they are easier to handle computationally, scale better to large models and datasets, and are easier to interpret. The prior knowledge used to construct logical network models frequently comes from reviewing the literature of a certain mechanism, disease or signaling pathway, and may be summarized in a database like STRING, Reactome or WikiPathways (Joshi-Tope et al., 2005; Kutmon et al., 2016; Rigden et al., 2016; Szklarczyk et al., 2017).

Logical models can be used to model stochastic processes. Probabilistic Boolean Networks (Shmulevich et al., 2002) have been introduced to simulate logical models in the presence of uncertainty, as they allow combining multiple Boolean networks with the respective continuous selection probabilities in one mathematical model. They have successfully been applied to the modeling of biological regulatory networks (Trairatphisan et al., 2013). This framework can be generalized to Dynamic Bayesian Networks (DBNs), a general class of models that includes Hidden Markov models and Kalman filters (Murphy, 2002), and can be used to represent the same joint probabilities between variables. In a graphical model of a DBN, the values of the different nodes represent the probabilities for randomly chosen molecules to be in an active state, while the edges represent the probabilities of the parent nodes to activate their targets. Network update is performed according to the laws of probabilities.

There is, however, a number of impediments to successful biomolecular modeling. Firstly, the prior knowledge used to build the model could be inaccurate, or more frequently, incomplete, or both. In other words, compared to the true network, databases likely contain additional edges, as well as miss others. Secondly, the information contained in databases is often generic, collected across cell types, genetic backgrounds, and experimental conditions. Given an interaction graph and a series of contexts (cell types, patients), the task of determining which interactions are context-specific and which ones are context-independent rapidly becomes intractable. This task is however essential to reduce the model complexity, as overly complex models are prone to overfitting (thus less generalizable), computationally expensive, and might be less interpretable than simpler ones. In addition, identification of the most variable model parameters between contexts has the potential to be directly informative about the mechanisms at play and help draw parallels between contexts.

Inter-patient variability is an important factor for many diseases, and in particular cancer. Intra-tumor heterogeneity has been recognized for a long time (Fidler et al., 1982) and it has been established that the heterogeneity of cell lines isolated from different patients spans the genomic, epigenetic, transcriptomic, and proteomic levels, resulting in large phenotypic differences, even within the same tissue of origin (Hoadley et al., 2014). Additionally, the patients' own genetic backgrounds and the tumor micro-environment also play a role in increasing the heterogeneity of clinical responses (Zhou et al., 2008; Marusyk and Polyak, 2011; Junttila and De Sauvage, 2013). However, recent successes in matching a biomarker with the sensitivity to certain targeted anti-cancer therapies, notably in the case of HER2-overexpressing breast cancer (Vogel et al., 2002), EGFR-mutated non-small-cell lung cancer (Lynch et al., 2004), BCR-ABL fusions in chronic myelogeneous leukemia (Sherbenou and Druker, 2007), and BRAF^V600E^-mutant melanoma (Bollag et al., 2010) suggest that the general approach of targeting specific mechanisms in subsets of patients harboring functionally similar tumors is clinically promising.

A number of methods have been devised for the general task of variable selection. Various methods rely on the intuitive notion of comparing models comprising different subsets of the independent variables (Hocking, 1976). This strategy is however problematic for several reasons. Firstly, the number of possible subsets grows very fast with the number of variables, leading to the infeasibility of testing them all. Secondly, repeatedly optimizing a model structure using the same dataset violates the central assumptions of the *F*-tests or χ^2^-based statistics used for comparisons, which are designed to test a single hypothesis. Strategies like forward-selection, backwards elimination, or combinations of both are consequently affected by numerous problems, notably biased parameter estimation and artificially low *p*-values (Harrell, 2001; Burnham and Anderson, 2002).

Fitting an overspecified model first and clustering the parameters in a second step is not a sound method to achieve sparsity, as the parameter estimates might not be stable, resulting in inaccurate clustering. Furthermore, the two objectives are not coupled, which is problematic: a small difference between the values of two parameters might or might not be supported by the data. It makes more sense to specify our assumptions about the distribution of the parameter values as part of the objective function. Regularization is a technique for adding prior information to a regression problem. It consists in adding to the loss function a function of the parameters alone. More formally, when attempting to learn the parameter set θ from dataset *X* = [*x*_1_, *x*_2_, …, *x*_*n*_] with a model *M*, the objective function *O* takes the form:

(1) O=f(M(X,θ),X)+λg(θ)

where *f* is the loss function, for example the sum of squared errors. The hyperparameter λ is used to balance goodness-of-fit with the regularization objective *g*(θ). The most common form of regularization is the Tikhonov regularization (Tikhonov, 1963), also called *ridge regression*, which materializes the assumption that small model parameters are more probable than larger ones. Also called the *L*_2_ norm, the Tikhonov regularization term takes the form:

(2) $$g(\theta )=\sum_{j=1}^{T}{\left(\theta_{j}\right)}^{2}$$

where *T* is the number of parameters of the model. The *L*_2_ norm is used to impose a penalty on large parameter values. Its popularity is due to the fact that the function is convex, continuous and differentiable everywhere, and is therefore well adapted to gradient descent optimization. It is mostly used in predictive models to avoid overfitting and produces models that are more generalizable. Because the gradient of this function becomes very small around zero, Tikhonov regularization does not achieve sparsity under most conditions and therefore does not perform variable selection, however this can be solved by the use of thresholds.

Intuitively, the most sensible sparsity constraint should be the *L*_0_ norm, or the cardinality of the non-zero parameter set:

(3) $$g(\theta )=\sum_{j=1}^{T}1_{\left(\theta_{j}\neq 0\right)}$$

where 1_(*C*)_ is the *indicator* function, and is equal to the number of cases where condition *C* is true. However, this is usually not feasible in practice, as this function is discontinuous and cannot be used in many optimization algorithms. A good approximation is the *L*_1_ norm, which sums the absolute values of the parameters, without squaring them:

(4) $$g(\theta )=\sum_{j=1}^{T}\left|\theta_{j}\right|$$

The *L*_1_ norm, or LASSO (Tibshirani, 1996) can be used to reduce the size of a model by efficiently removing variables (i.e., set their coefficients to zero) which contribute the least to the model. Importantly, by screening a range of regularization parameter λ, it is possible to order the variables according to their importance. It is natural to use it then, for contextualizing models of biological systems with measurements from different contexts to point to their differences. Different approaches have used the *L*_1_ norm to contextualize network models of signal transduction in mammalian cells. However the assumption is either that there is no relationship between the different cell lines (Eduati et al., 2017; Lucarelli et al., 2018), or that the differences to the mean value should be minimized (Merkle et al., 2016). While the latter works in the case of only two cell lines, it does not when comparing more. The reason is that heterogeneity between cell lines is expected, and we know that different mechanisms are at play in a given experiment. By penalizing any difference, such regularization does not allow parameters to have two or more possible values. However, cancer-related perturbations to molecular interactions occur in discrete steps. Driver mutations often result in the complete loss of the function of a certain protein, for example p53, or constitutive enzymatic activity, for example the common mutation of genes of the RAS family (Kandoth et al., 2013). The desired regularization should therefore penalize differences between contexts but allow for a structure in the parameter space. While a number of methodologies exist (Dondelinger et al., 2012; Hill et al., 2012) to regularize network models of signaling pathways for time-stamped data, in that case the structure of the prior on the parameter space is known, as time is oriented. We propose that the correct assumption for analyzing perturbation data from multiple cell lines, cell types, or across patients, is that network parameter values would form *clusters* corresponding to the most common signaling deregulations. However, methods to efficiently identify the parameters of a biological model and cluster them at the same time are missing.

The general problem of regularizing a model toward a specific, although unknown, structure has been investigated before. The vast majority of the proposed methods combine *L*_1_ and *L*_2_ norms in various ways. Group LASSO (Yuan and Lin, 2006) was introduced to allow the selection of entire groups of variables. This was then extended to a hierarchical selection of nested groups of variables (Zhao et al., 2009), partially overlapping groups of variables (Jacob et al., 2009), and to the induction of sparsity within groups by penalizing for pairwise differences between coefficients of variables belonging to the same group, with the OSCAR algorithm (Bondell and Reich, 2008) and the clustered LASSO (She, 2010). Later Simon et al. proposed the sparse group LASSO (Simon et al., 2012), a modification of the *elastic net* criterion proposed by Zou et al. which combines the *L*_1_ and *L*_2_ norms (Zou and Hastie, 2005). The fused LASSO (Tibshirani et al., 2005) is applicable when there is a natural ordering in the model variables, like time-stamped or spatially organized data. Several groups have tried to decouple the steps of clustering and model fitting, either by considering all possible clusters (Jenatton et al., 2011) or by applying first hierarchical clustering based on the measurements covariance matrix (Bühlmann et al., 2013).

While these approaches have proven useful in some cases (Zhang et al., 2014; Steiert et al., 2016), they do not apply well to the case of regulation networks, because group zero-sparsity (removal of entire groups of variables, as opposed to within-group sparsity) is not necessarily desired, except in the case of network pruning. We therefore implemented a regularized version of the objective function of the FALCON toolbox (De Landtsheer et al., 2017), to lower the degrees of freedom of the model by encouraging the grouping of model parameters across contexts, regardless of the number of groups. This can be achieved by detecting anomalies in the parameter values distribution, assigning a penalty to groups of values more alike the reference null distribution. In our case (Bayesian Networks), the uniform distribution [0−1] is assumed to better represent the prior of uncorrelated parameter values, as they are usually interpreted as probabilities. Under different modeling formalisms, other distributions would be more appropriate, for example for ODE-based or constraint-based models. We show how the penality correlates with other measures, with unsupervised clustering, and we demonstrate the use of regularized fitting, first on a small synthetic network model, then with biological data.

## 2. Methods

### 2.1. Algorithm

We propose a measure of uniformity of the parameter values distribution modified from previous work in the field of quasi-random sequences (Sobol, 1976). Given a parameter space **P** and *N* parameter vectors with *T* parameters θ_1_, θ_2_, …, θ_*N*_, with $\theta_{n}=\left\{\theta_{n}^{1},\theta_{n}^{2},\ldots ,\theta_{n}^{T}\right\}$, we compute for each *t* ∈ *T* the average absolute deviation from the expected local density of points *D*_*t*_ with:

(5) $$D_{t}=\sum_{R\in P}\left|1_{\left(\theta_{n}^{t}\in R\right)}-Vol(R)\right|$$

for all rectangles **R** = [*a*_1_, *b*_1_] × [*a*_2_, *b*_2_] × … × [*a*_*T*_, *b*_*T*_] such that 0 ≤ *a*_*i*_ ≤ *b*_*i*_ ≤ 1, and with *Vol*(**R**) being the volume of the *T*-dimensional rectangle **R**.

(6) $$Vol(R)=\prod_{i}b_{i}-a_{i}$$

The first term in Equation 5 represents the *observed* density of points, while the second one represents the *expected* density. These two quantities are equal in the case of perfect uniformity. We then define the *uniformity* *U* of the parameter vector as the inverse of the average deviation over the *T* parameters:

(7) $$U_{t}=\frac{T}{D_{t}}$$

and the *uniformity* of an entire model parameter set as the average over all vectors:

(8) $$U=\frac{1}{N}\sum_{i=1}^{N}U_{i}$$

**Figure 1:**
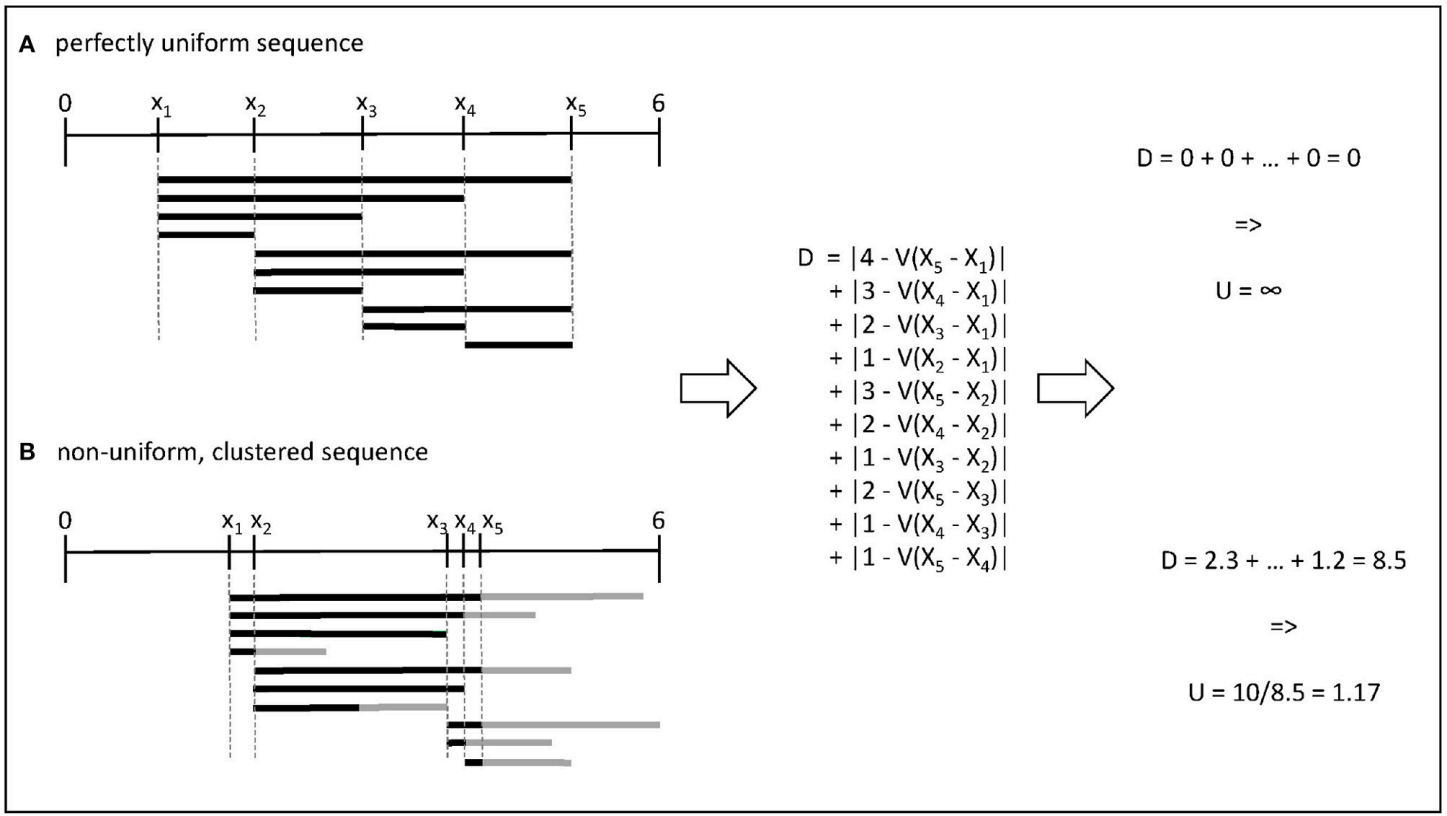
Illustration of the computation of uniformity for two sets of 5 parameter values within the range [0, 6]. **(A)** In the first case, all pairwise distances are equal to the expectation given the rank of the value in the set. **(B)** In the second case, the gray bars indicate the differences compared to the expected density in a given interval.

In one dimension, this metric has an intuitive interpretation, as shown in Figure 1: when parameter values are as different as they could be, the expected difference between any two values can be calculated from their relative rank in the set. For example, the distance between two successive observations is $\theta_{n}^{t}-\theta_{n-1}^{t}=1/N$. When values cluster together, they create windows in which the local density is either higher or lower than this expected value. Note that in one dimension, the rectangles **R** are equivalent to the distance between the points, and to the convex hull of these points, while it is not true in higher dimensions.

### 2.2. Uniformity as a penality in regularized fitting

We analyze the sensitivity of our new metric to the amount of structure in sets of model parameter values by computing it for a large number of sets of uniformly, independently distributed random values. We compare uniformity with the standard deviation, with the results of the Kolmogonov-Smirnov (K-S) (Massey, 1951) and Anderson-Darling (A-D) tests (Anderson and Darling, 1954), and with the sum of pairwise distances. The two non-parametric statistical tests aim at comparing the empirical distribution of the values in the set with a reference distribution, in this case the uniform distribution. The sum of pairwise distances is used in Bondell and Reich (2008) and She (2010), the standard deviation is examplative of measures of spread around a single value, like in Merkle et al. (2016). In addition, we compute for each set the optimal number of clusters (explaining 90% of the variance) using the k-means algorithm and the elbow method (Ketchen and Shook, 1996). Using the inferred number of clusters, we compute the sum of intra-cluster distances. We performed this comparison with 10^4^ vectors. Also, to assess the usability of this metric for large-scale computations, we compare the running time of the different computations for sets of size 10, 20, and 40, simulating models with increasing number of contexts.

To illustrate that the use of uniformity as a penalization in an objective function results in the convergence of parameter values into clusters, we iterate a gradient descent process for random sets of uniformly, independently distributed random values. This is equivalent as optimizing a null model using uniformity as a regularizing function, and shows the effect of this penalization in the absence of data. We used gradient descent (using empirical gradients and the interior-point method) with a learning rate of 10^−3^, collect the shape of the set over 100 updates, and we compare with the centroids of the k-means clustering. All computations were done using Matlab 2017a on a standard desktop computer which specifications are detailed in section 2.3.3.

### 2.3. Modeling experiments

Modeling experiments in this paper used the toolbox FALCON (De Landtsheer et al., 2017), a Matlab-based versatile tool to contextualize logical models of regulatory networks. Briefly, FALCON uses a Dynamic Bayesian framework (Lähdesmäki et al., 2006) in which Boolean operations are explicitly defined as arithmetic, continuous logical functions. FALCON emulates a Probabilistic Boolean Network with user-defined topology and uses experimental data from perturbation assays to optimize the weights of the network, which represent the relative activating and inhibiting influences of the network components with respect to the logical functions. For the large-scale analysis of biological data, we used a custom implementation of FALCON running on a high-performance computing platform which specifications are detailed in section 2.3.3.

(9) $$O=\frac{1}{n}\sum_{i=1}^{n}{\left(Y_{i}-\hat{Y_{i}}\right)}^{2}+\lambda U(\theta )$$

where *Y* is the vector of measurements for the observed nodes, Ŷ is the vector of corresponding predictions and *U*(θ) is the uniformity of the parameter set θ across contexts, as defined by Equations 5–8 above, with λ being a scalar that controls the relative contribution of the penality to the objective function. The code and data files used for both the synthetic model and the biological example are available at the address https://github.com/sysbiolux/FALCON. Additional driver scripts are provided in the Supplementary Materials.

#### 2.3.1. Synthetic toy model

**Figure 2:**
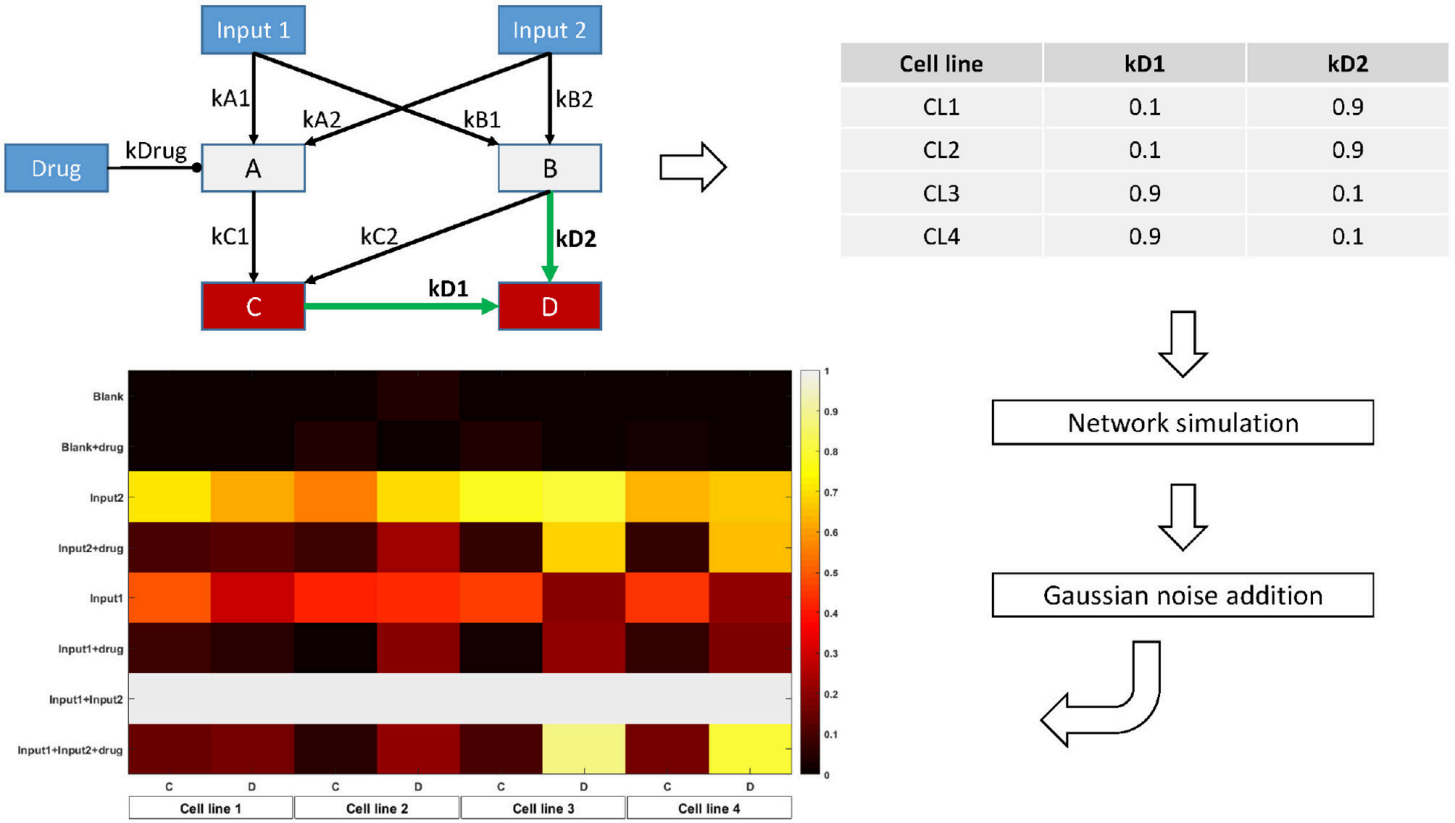
Overview of the toy model design. The topology is parametrized in order to display two-by-two similarity between cell lines. For each cell line, the Bayesian Network is simulated with the corresponding parameter values for 8 different combinations of the input nodes values. Random Gaussian noise is added to the values of the two output nodes C and D, simulating biological measurements. The heatmap shows the final node values for each condition, cell line, and node.

In order to assess the use of our regularization scheme for finding context-specific parameters, we design a simple toy model with 7 nodes and 9 edges. Two of these nodes are inputs, while two others are measured. We set the model parameters differently for four conceptual cell lines, in such a way that while most parameters are conserved, some would be different, and shared across several (but not all) cell lines. Figure 2 shows a graphical representation of the network, the values chosen for the model parameters, and the final synthetic data used for model fitting.

To realistically simulate biological data, we use our toy model to generate synthetic steady-state data for the measured nodes by simulating the network with different combinations of values for the input nodes, thereby mimicking a designed, perturbation experiment. We simulate noise in the data by adding a two-component gaussian perturbation around the theoretical value, as explained in Supplementary Methods. The magnitude of the perturbation was chosen to reflect the signal-to-noise ratio of typical biological measurements, for example phosphoproteomics or microarray data.

#### 2.3.2. Biological dataset

To show the usefulness of our approach in a biological setting, we reanalyze the dataset from Eduati et al. (2017), in which the authors measured 14 phosphoproteins under 43 different perturbed conditions (combinations of 5 stimuli and 7 inhibitors) in 14 colorectal cancer cell lines. Using CellNetOpt (Terfve et al., 2012), they contextualized independent logical ODE models (Wittmann et al., 2009) for each cell line, and proceed to train a statistical model using the cell-specific parameters to predict the responsiveness of the cell lines to a panel of drugs. This study provides an example of the use of system-level analyses to gain understanding of functional properties that cannot be inferred by genomic features alone. We normalized the data (*log*_2_ difference compared to control) linearly to the [0−1] range across cell lines.

Logical ODE models like the one used by Eduati et al. rely on a transformation of the discrete state-space of Boolean models into a continuous one, in such a way that Boolean behavior is preserved on the vertices of the unit cube, i.e., when the inputs are in {0, 1}. While there are many such possible transformations (Wittmann et al., 2009), the authors chose to use normalized Hill cubes, which are sigmoidal functions of the inputs. The strength of such an approach is the ability to take into account the non-linear 'switch-like' nature of molecular interactions, however at the expense of doubling the number of free parameters (Hill functions are defined by a threshold and a slope). In contrast, our approach uses maximum one parameter per interaction and is restricted to linear relationships, which ensures coherence with the laws of probabilities. To infer the DBN model corresponding to the logical ODE model proposed by Eduati et al., we kept the original topological information, but defined the update function for each node by a multivariate linear function of its parent nodes. In our framework, if two nodes *A* and *B* are both activators of a third node *X*, we have for each time-step *t*: *X*_*t*_ = *k*_*A*_*A*_*t*−1_ + *k*_*B*_*B*_*t*−1_ with probabilities 0 ≤ *k*_*A*_ ≤ 1 and *k*_*B*_ = 1 − *k*_*A*_. Similarly, if a node *X* is activated by node *A* but inhibited by node *B*, we have *X*_*t*_ = *A*_*t*−1_*k*_*B*_(1 − *B*_*t*−1_) with probability 0 ≤ *k*_*B*_ ≤ 1.

We used the phosphoprotein data to fit the probabilities for each interaction simultaneously for all cell lines. The complete model comprised 363 nodes and 1106 parameters. The objective function included a penality computed from the average uniformity of the parameters across cell lines, according to Equations 5–8. We optimized 49 models, varying the hyperparameter λ from 2^−20^ to 2^5^, and we recovered the optimal parametrization for each cell line in the form of regularization paths. We used the value of 0.01 as threshold for deciding if two parameters should be merged into a single one. For each value of the regularization strength λ, we computed the mean squared error (MSE) and the number of different parameters *P* in the regularized model, and from these calculate the Bayesian Information Criterion (BIC), which we calculate as *Nlog*(*MSE*) + *log*(*N*)*P*, with *N* the number of individual points in the dataset. Lower BIC values indicate models with favorable balance between goodness-of-fit and model complexity (Schwarz, 1978; Burnham and Anderson, 2004).

We selected the model with the lowest BIC for further analyses. We grouped cell line-specific parameters together using the above-mentioned threshold, and re-optimized the model using the obtained topology without the regularization term, in order to obtain unbiased parameter estimates. We performed hierarchical clustering with 1000 bootstrap resamplings on the parameter values using WPGMA and euclidian distance.

Furthermore, we investigated whether the recovered parameter values are associated with drug sensitivity. We downloaded the IC50 values for the 14 cell lines and 83 drugs directly targeting either one of the network's nodes or a target used in clinical practice to treat colorectal cancer from the Genomics of Drug Sensitivity in Cancer database (www.cancerrxgene.org). We computed the linear regression models between each drug and each of the 31 parameters which showed high variability between cell lines (CV ≥10%). The F-statistic was used to compute a *p*-value for each test, and *q*-values were computed from these, using the Benjamini Hochberg procedure to control the False Discovery Rate.

#### 2.3.3. Materials

- Hardware
  - Synthetic model: standard desktop computer equipped with an Intel Xeon E3-1241 CPU clocked at 3.50GHz and 16GB of RAM under Windows 7
  - Biological example: high-performance computing platform with 49 nodes running Matlab2017a, each node consisted of one core of a Xeon-L5640 clocked at 2.26GHz with 3GB RAM

- Software
  - Matlab 2017a (Mathworks, Inc.)
  - FALCON toolbox (https://github.com/sysbiolux/FALCON)
  - Optimization Toolbox (http://nl.mathworks.com/products/optimization/)
  - Parallel Computing Toolbox (http://nl.mathworks.com/help/distcomp/)
  - Bioinformatics toolbox (http://nl.mathworks.com/help/bioinfo/) (optional)

## 3. Results

### 3.1. Uniformity as a measure of structure

**Figure 3:**
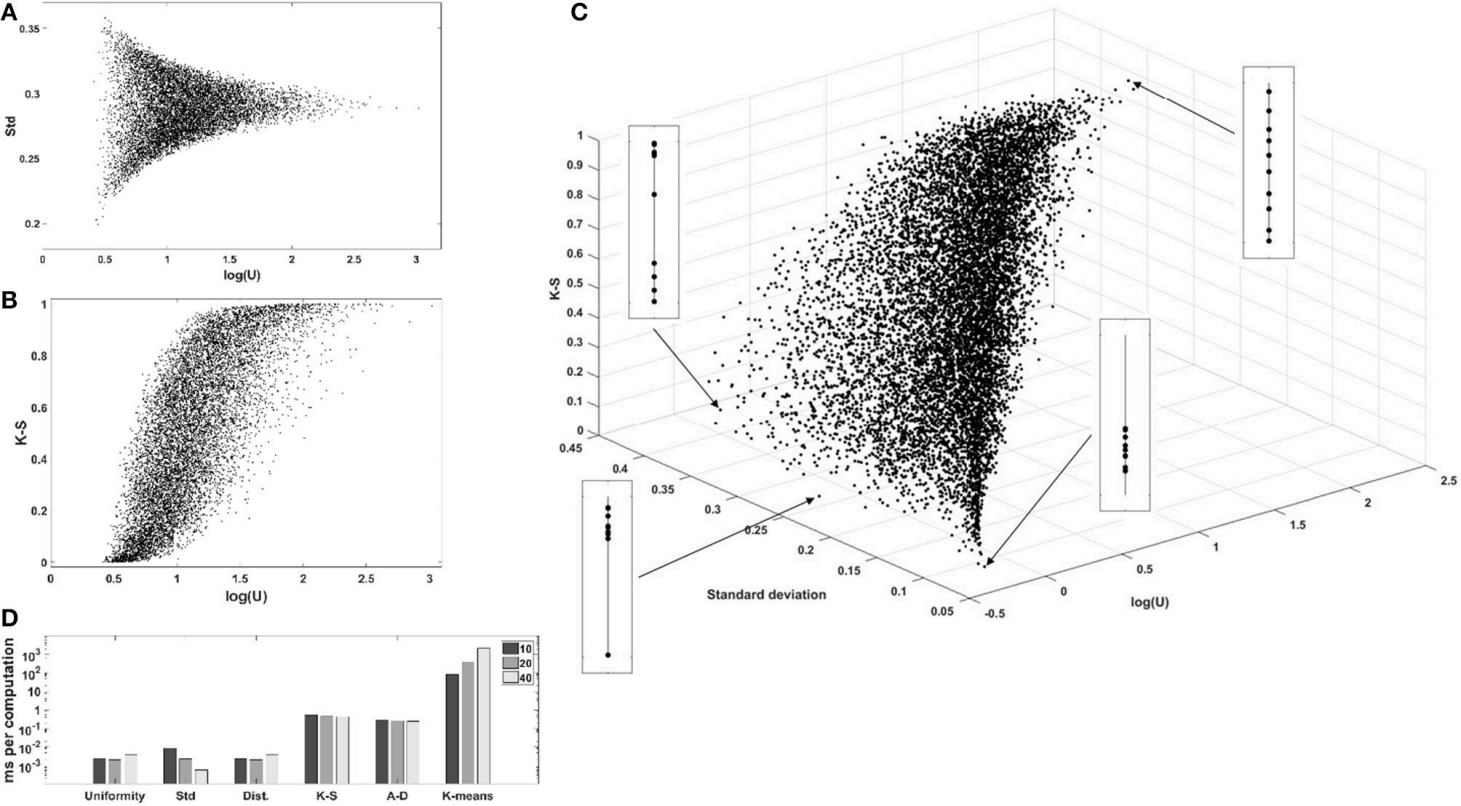
Evaluation of uniformity *U* as a measure of structure, for 10^4^ one-dimensional sets of 10 values. **(A)** Comparison with standard deviation. **(B)** Comparison with the *p*-value of the K-S test (similar results were obtained with the A-D test). **(C)** 3D-scatterplot of uniformity, standard deviation and K-S *p*-value. **(D)** Computation times for the different metrics. log(U), log_2_(uniformity); Std, standard deviation; Dist, sum of pairwise distances; K-S, *p*-value of the Kolmogonov-Smirnov test; A-D, *p*-value of the Anderson-Darling test; K-means, k-means clustering, number of clusters determined with the elbow method.

We computed the uniformity *U*, the standard deviation, the sum of pairwise distances, the K-S statistic, the A-D statistic, and the optimal number of clusters using the k-means algorithm and the elbow method, for 10^4^ one-dimensional sets of uniformly, independently distributed random values. The complete correlation plots are presented in Supplementary Materials. We always show uniformity *U* on the logarithmic scale. Figure 3A shows the relation between uniformity and the standard deviation, while Figure 3B shows the correlation between uniformity *U* and the *p*-value of the K-S test. Similar results were obtained with the A-D test. The relationship between uniformity, the standard deviation, and the K-S *p*-value are further explored in Figure 3C, and the computation times are compared in Figure 3D.

Firstly, *log*(*U*) is positively correlated with the *p*-value of the K-S and A-D non-parametric tests evaluating the distance to the reference uniform distribution, showing that low uniformity is indicative of structure. Secondly, the comparison with the standard deviation shows that low-uniformity sets can have drastically different standard deviations, but that the inverse is not true. This is explained by the fact that sets with tightly clustered values will nevertheless be spread around the global average if there is more than one cluster. Figure 3C shows a 3D plot of uniformity, standard deviation, and the K-S *p*-value and illustrates the point that simple measures of spread are not adapted to the regularization of a set of parameter values if the ground truth is that there is more than one cluster. The figure also displays a graphical representation of the 10 values in the set for four chosen sets, to show that low-uniformity sets correspond to clustered values (with low K-S *p*-values) while low standard deviation is associated with single clusters.

One important argument for choosing a metric in a regularized optimization problem might be its low computational cost. Comparison of the running time for uniformity with other metrics shows that the new metric can be computed very efficiently (Figure 3D), several orders of magnitude faster than the non-parametric tests or the clustering algorithm. This low computational cost makes is well adapted to the repetitive computations characteristic of gradient-descent optimizations.

**Figure 4:**
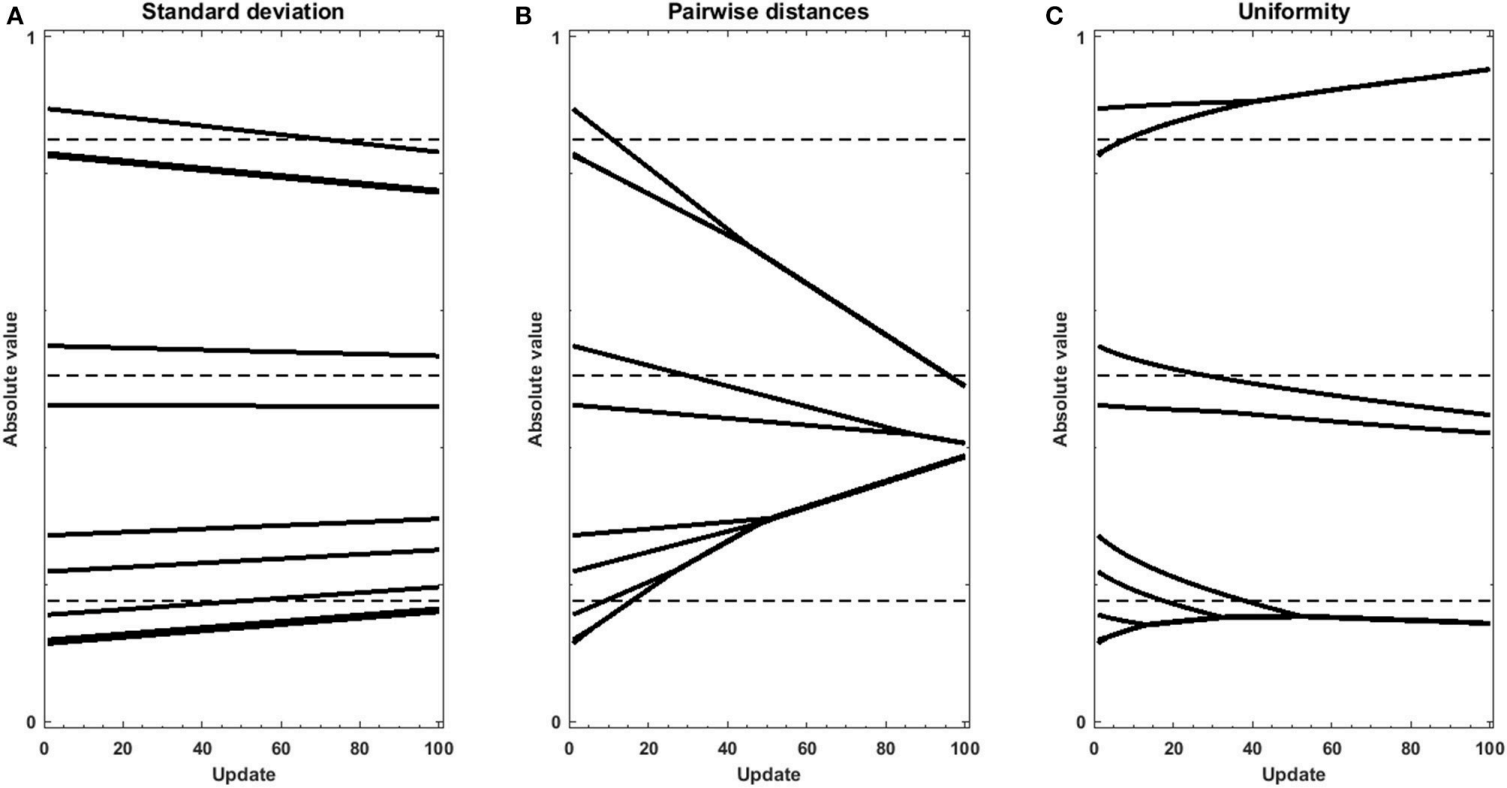
Gradient descent trajectories for a set of randomly uniformly distributed values displaying a certain level of structure, using different metrics as objective function: **(A)** standard deviation, **(B)** sum of pairwise distances and **(C)** Uniformity *U*. The dotted lines show the values of the centroids of clusters as determined by the k-means + elbow method for the original vector.

In addition, we performed experiments using gradient descent either with the standard deviation, sum of pairwise distances, or uniformity *U* as an objective function on sets of randomly, uniformly distributed random values. Using the regularization objective as the objective function, without data or model to produce an error function, helps understanding the effect of regularization when signal is low in the data. The traces in Figure 4 reveal the strength and direction of the bias applied on each value in the set in the absence of cost function. Penalizing on the standard deviation results in a homogeneous pull toward the average value (Figure 4A), which does not accomplish the goal of forming clusters. Using the sum of pairwise distances, in turn (Figure 4B), results in grouping of values together, however the clusters themselves are still pulled together. In contrast, the traces in Figure 4C show that using uniformity *U*, the values form a number of groups, but that these groups are more stable. This is due to the fact that the computation of uniformity *U* measures local density both below and over the expected value, which means that not only clusters but also voids produce low-uniformity sets. As a result, once values with all clusters have merged, the average of the different clusters remain very similar in number and value to the centroids of the k-means clustering.

### 3.2. Toy model

**Figure 5:**
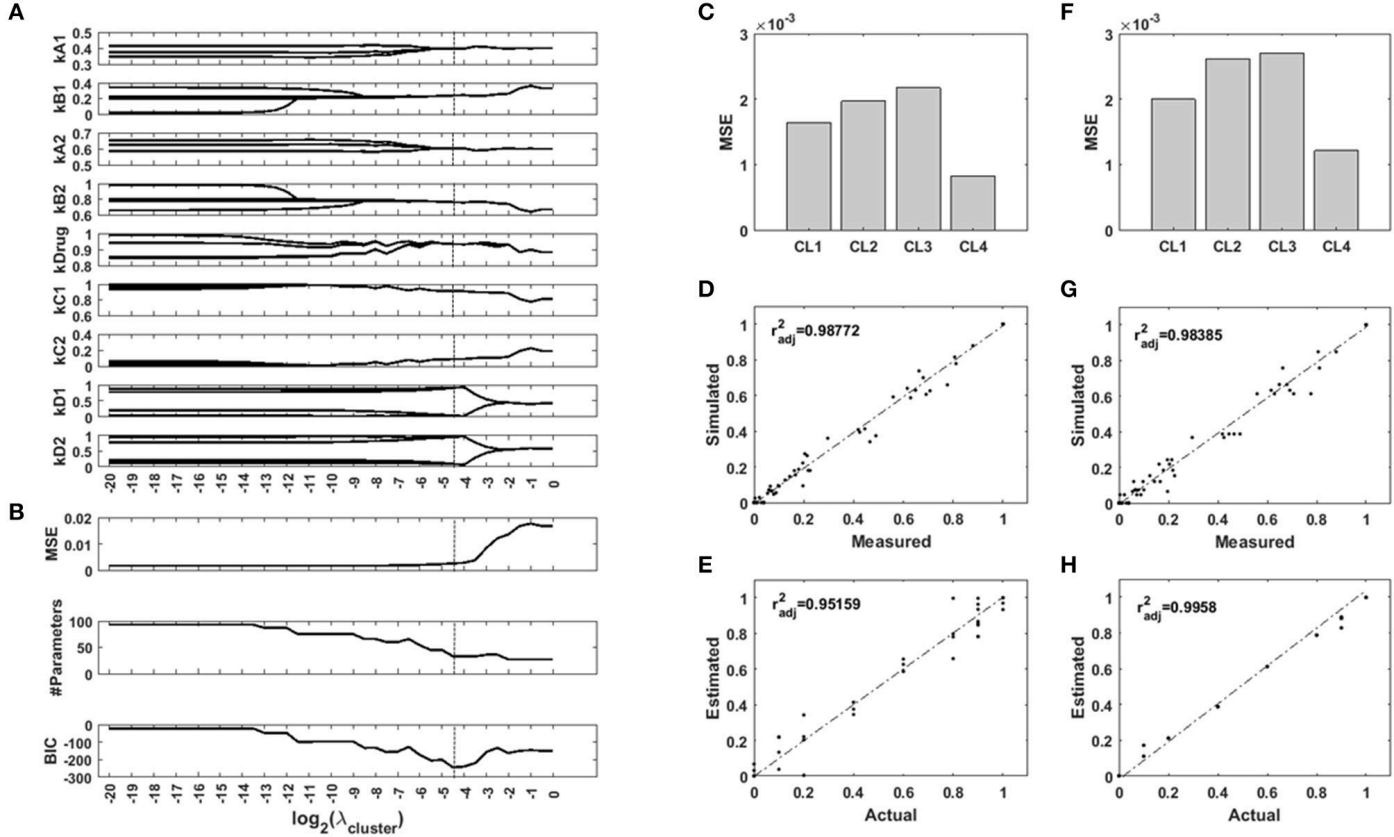
Results of the synthetic toy model analysis. **(A)** Regularization paths for each parameter of the network model. When regularization strength increases, values accross the four contexts are encouraged to merge. **(B)** Mean squared error (MSE), number of different parameters of the model, and Bayesian Information Criterion (BIC) for different regularization strengths. **(C,D,E)** Unregularized model. **(F,G,H)** Sparse model. **(C,F)** MSE for the four contexts with both models. **(D,G)** Comparison of the simulated node values with the measurements for both models. **(E,H)** Comparison of the inferred parameter values with the ground truth for both models. $r_{adj}^{2}$: adjusted Pearson's correlation coefficient.

To test the ability of a regularization function using uniformity *U* to recover context-specific parameters of a network model, we generated an example Bayesian Network which we parametrized for four different imaginary contexts. In our example, the contexts are cell lines, and their regulatory network are identically parametrized two by two. We used the network to generate measurements for two of the nodes while two other nodes were controled. We added noise to this synthetic data to simulate background noise and normaly distributed measurement errors. We used the toolbox FALCON to contextualize the network for the four cell lines, with and without regularization based on the uniformity *U* of the set of parameter values. We screened 41 values of the hyperparameter λ. The computations took a total of 220 seconds on a standard desktop computer. The results are presented in Figure 5. The regularization paths in Figure 5A show the optimal parameter values over a range of regularization strengths λ. The unregularized model is parametrized differently for each cell line, and the regularization induces a grouping of the parameters values across cell lines. However, this clustering occurs at different values of λ. As regularization strength increases, so does the error of the model (Figure 5B), while the number of unique parameters in the model decreases as they are merged together. We used the Bayesian Information Criterion to balance goodness-of-fit with model size and identified λ = 2^−4.5^ as the best model configuration. **Figures 5D,F** show the fitting cost for each cell line for the unregularized model and the regularized one, respectively. **Figures 5D,G** show the correlation of the simulated values with the measurements, for the unregularized model and the regularized one, respectively, and **Figures 5E,H** show the correlation of the inferred parameter values with the real values for the unregularized model and the regularized one, respectively. Together, these results show that while the new model displays a higher MSE, the inferred parameters are much closer to the ground truth. Regularization transfers a portion of the variance from the parameters back to the data, and so decreases the part of the error on the parameter estimates due to noise. More importantly, the grouping of the samples is easily recovered (Supplementary Figure S2), which also carries information: the cell lines are identical two-by-two.

### 3.3. Biological dataset

**Figure 6:**
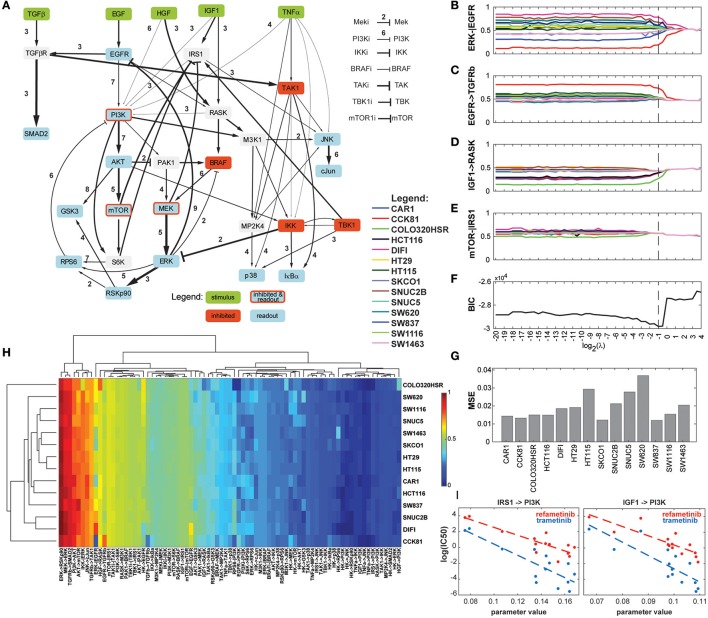
Results of the analysis of the biological dataset. **(A)** Optimized network topology (adapted from Eduati et al., 2017). The width of the arrows represents the median parameter value across the 14 cell lines, with wider arrows corresponding to the most active interactions. The number next to the arrows is the number of clusters that the 14 cell lines form for the optimal regularization strength. **(B–E)** Regularization paths for four chosen interactions, showing decreasing amounts of cell line-specificity. **(F)** BIC (Bayesian Information Criterion) path. **(G)** MSE (Mean Squared Error) for the 14 cell lines for the optimized model. **(H)** Heatmap of the values of the 79 parameters for the 14 cell lines. Dendrograms were produced with WPGMA using euclidian distance. **(I)** Correlation between two PI3K-related parameters and sensitivity to two MEK inhibitors. Left: IRS1-PI3K; refametinib: *r*^2^ = 0.737, *p*-value = 0.133; trametinib: *r*^2^ = 0.671, *p*-value = 0.176; Right: IGF1-PI3K; refametinib: *r*^2^ = 0.701, *p*-value = 0.146; trametinib: *r*^2^ = 0.652, *p*-value = 0.185.

In order to assess the applicability of our new method of regularization to uncover context-specificity in a realistic modeling setting, we reanalyzed the data from Eduati et al. (2017) using a Dynamic Bayesian Network adapted from the topology of the ODE model. The dataset comprised 8428 datapoints (14 phosphoproteins for 14 cell lines under 43 experimental conditions). We screened 49 values for the hyperparameter λ. The computation time was 1,761 h, or 42 h when parallelized among 49 computing cores. The results are presented in Figure 6. Minimum BIC was reached when λ = 0.5, which corresponds to a model in which 26 of the 79 network parameters can be parametrized identically for all cell lines, and the remaining ones can be organized in 2–9 groups. Overall, the most variable parameter across cell lines is the ERK-EGFR negative feedback (Figures 6A,B). Notably, interactions relating to the PI3K/Akt/mTOR axis, to the JUN pathway, and to p38 regulations showed relatively high heterogeneity compared to the crosstalks between them. A number of interactions reveal differential parametrizations for certain cell lines, for example CCK81 in the case of TGFRβ activation by EGFR (Figure 6C), or COLO320HSR in the case of RASK activation by IGF1 (Figure 6D). Figure 6E shows an example of regularization path where no cell line specificity is left in the model with the optimal topology. In addition, many interactions (narrower arrows in Figure 6A) show very low values for all cell lines, suggesting that they do not play an important role in this experiment. The complete set of 79 regularization paths is presented in the Supplementary Materials. The changes in BIC are shown in Figure 6F, displaying a marked minimum around the value 0.5. The goodness-of-fit was similar for all cell lines, with MSE values ranging from 0.018 to 0.035 (Figure 6G). While these results are in line with the ones reported in the original study, it should be noted that in our final model, the role of TAK1 is less prominent, a fact that can be explained by the difference of modeling paradigm. Indeed, while in Eduati et al. (2017) TAK1's *node responsiveness* parameter τ is extremely low for all cell lines while edges from and to TAK1 are quite variable, our modeling framework considers all nodes equally responsive, and as a consequence low TAK1 activity is represented by low edge parameter values.

Figure 6H shows a heatmap of all model parameters for all cell lines. The dendrograms show the clustering of model parameters and cell lines based on their parameter values. We chose WPGMA to perform hierarchical clustering using the euclidian distance between parameter vectors, with 1000 bootstrap replicates. The support for the nodes in the cell line dendrogram are indicated as percentages. Interestingly, cell lines HT29 and HT115 cluster strongly together, while they are highly dissimilar in their genomic alterations. In general, we noted a poor correlation between the genomic and functional pattern over this set of cell lines, a fact already noted in the original study. Cell lines COLO320HSR and CCK81 are the cell lines functionally most unlike the others. This is also visible in the raw data (see Supplementary Materials), notably in the amplitude of the Akt/PI3k/MEK activations.

Next, we explored the possible associations between the 31 most variable model parameters and sensitivity to 83 chosen drugs. The 25 most statistically significant of these linear associations are presented in the Supplementary Materials. While no parameter-drug pair shows strong significance (most likely due to the high number of hypotheses tested), we noticed a pattern in which some parameters seem to correlate with sensitivity to MEK inhibitors. Figure 6I shows that the parameters relating to PI3K activation by IRS1 and IGF1R are inversely correlated to the log(IC50) of refametinib and trametinib, two known MEK inhibitors.

## 4. Discussion

We propose a new measure of the degree to which sets of values are clustered around an unknown number of centers. We use this new metric, called uniformity *U*, as a penalization in the objective function of models of signal transduction. Previously, regularization applied to the parameters of such models have assumed either that parameter values would be mostly identical across the different studied contexts (using measures of spread), and looked for departures from this assumption for context-specific parametrizations, or that the parameter values would change in correlation with another, known variable between samples (e.g., smoothly over time). While these assumptions make intuitive sense, they are probably not usable in the case of models of regulatory networks in a large number of cell lines. Indeed, functional relationships between molecules in cells, like enzymatic rates and binding strengths, usually exist in a small number of versions for a specific interaction. Because we do not expect these properties to change along a continuum but in a discrete way, it is natural to assume that model parameters of a regulatory network display the same type of structure. Our method efficiently reduces the complexity of network models. In our toy model example, we decrease the number of parameters from 32 to 11, and correctly recover the fact that two groups of cell lines exist and should be parametrized differentially. In our biological example, we decrease the number of parameters from 1,106 to 272, without increasing the error disproportionately.

We show that this method is applicable to biological studies by re-analyzing the dataset from Eduati et al. (2017). Our analysis indicates that the most variable interactions relate to the PI3k/Akt/ERK axis, in particular the ERK/EGFR negative feedback. Interestingly, it has been shown that negative regulation of the MAPK pathway by ERK is a highly complex mechanism and comprises several components, many of which are affected by cancer mutations (Lake et al., 2016).

By performing hierarchical clustering on model parameters after fitting the data to the best model topology, we recover a grouping of the cell lines that correlates poorly with the genomic alterations. We hypothesize that this means we capture a degree of functional heterogeneity that cannot easily be explained by the cell lines' genomic features. Further indication that our network approach is able to recover phenotypical information that is not obvious in the raw measurements is provided by the pattern of relatively strong correlation between a number of parameters and sensitivity to several MEK inhibitors. This observation fits into the recent developments made in integrating network modeling approaches with advanced statistical modeling, where machine-learning methods have been used to successfully predict sensitivity to single drugs and to drug combinations (El-Chaar et al., 2014; Way et al., 2018). Further work is needed to quantify the merits of our regularization scheme when applied in such context.

Our key contribution is the demonstration that using a simple measure of parameter coefficients density inside the parameter space, it is possible to regularize a large network model and to efficiently group together model parameters for which the difference is not well supported by the data. By *de facto* removing part of the noise in parameter estimates, we are able to decrease model complexity. Furthermore, our regularization scheme is easily adaptable to stronger or weaker priors. Equation 8 can be modified as follows:

(10) $$U=\frac{1}{N}\sum_{i=1}^{N}U_{i}w_{i}$$

with *w* being the set of relative weights for the different parameters. When *w*_*i*_ = 1∀*i*, all parameters are regularized with the same strength. This weighted average allows the specification of additional prior information, namely that the structural assumptions might not be true everywhere, or that our confidence in these assumptions might be stronger in some cases than others.

It is likely that in the near future, single-cell proteomic studies will provide ever-larger datasets, therefore challenging modeling formalisms and requiring them to adapt to larger number of features (Spitzer and Nolan, 2016). While statistical analyses have largely benefited from regularized parametrizations in the form of more predictive models, the current regularization objectives are not well adapted to the study of signaling networks.

A natural extension of this regularization scheme is to consider subsets of *M* parameters, corresponding to coherent parts of the model, like known signaling pathways. In that case, regularization will act simultaneously on the different constituent parameters of the pathway, and will allow the determination on cell line-specific pathway activity, a high-level information which is usually recovered by ontology-based pathway analysis. However, in such two-step analysis, the confidence for the different parameters is lost. In addition, ontology-based analyses use pathway knowledge from databases, thus suffer from their incompleteness and inaccuracy.

Finally, although we have demonstrated the applicability of this novel method to the study of regulatory networks with logical models, it would be straightforward to extend its use to other modeling environments. For example, systems of ODEs, which are often used to model regulatory networks, might benefit from the addition of a new kind of regularization, using the same methodology presented in this paper. More generally, regularization based on the uniformity of coefficients would in principle be applicable to any type of regression problem and therefore has the potential to be integrated in many analytical frameworks, and be relevant to advanced statistical analysis.

## Author contributions

SD conceived the study, conducted experiments, and wrote the manuscript. PL proposed critical improvements, conducted experiments, and helped editing the manuscript. TS supervised the study, improved the study design, the experimental design and the manuscript.

### Conflict of interest statement

The authors declare that the research was conducted in the absence of any commercial or financial relationships that could be construed as a potential conflict of interest.
