# Supplementary material for: Using Regularization to Infer Cell Line Specificity in Logical Network Models of Signaling Pathways
Source: Front Physiol. 2018 May 22;9:550. doi: 10.3389/fphys.2018.00550 (PMC5972629; doi:10.3389/fphys.2018.00550)
Supplement: Supplementary file 1 [file Presentation_1.PDF]

Supplementary Material: Using Regularization to Infer Cell Line Specificity in Logical Network Models of Signaling Pathways

Sébastien De Landtsheer, Philippe Lucarelli, and Thomas Sauter*
*Correspondence:
Thomas Sauter:
thomas.sauter@uni.lu

1 SUPPLEMENTARY METHODS

1.1 Synthetic Data

To demonstrate the usability of our method to infer the context-specificity of parameter values, we generated a synthetic model and corresponding dataset which we used to verify the ability of our pipeline to recover the correct topology. The usefulness of our method lies in the ability to compare the inferred parameter values across cell lines and to decide if the signal present in the data is sufficiently low to justify the decision to merge the parametrization of a certain model parameter into a single coefficient. For this reason, we added random errors in the measurements, in a way that mimics the error usually present in real biological measurements. The file *DataGenerating.xlsx* details the generation of the synthetic data and contains the actual values used for the simulations presented in the paper.

In short, we mapped the inputs of the model (which values are known) to the outputs, according to the logical network presented in Figure 2 (main paper), using arbitrary values for the parameters, in such a way that they are equal across the four example cell lines, except for one pair of parameters. We then modified the computed output values Y to obtain the noisy output values Y' as follows:

$$Y' = Y + |X_1| + X_2 \tag{S1}$$

with the random variables X_1 (representing the positive background noise) and X_2 (representing the measurement error):

$$X_1 \sim \mathcal{N}(0, \sigma_1^2), \text{ and } X_2 \sim \mathcal{N}(0, \sigma_2^2) \tag{S2}$$

where $\sigma_1 = \sigma_2 = 0.05$.

1.2 Algorithm pseudocode

We provide pseudocode to facilitate implementation of our methodology in a different environment. The following sequence computes the uniformity U for a parameter set X.

```
Data: column vector X
Result: scalar U
initialization;
sort X ascending;
L = length(X);
for c from 1 to L-1 do
  | for cc from c+1 to L do
  |   | U = U + abs((X(cc) - X(c)) - (cc-c)/L)
  | end
end
U = L/U;
```

Algorithm 1: Computation of uniformity

2 SUPPLEMENTARY FIGURES

We provide to the reader additional figures: Figure S1 shows the complete correlation plot matrix for the different metrics used in the evaluation of the regularization function. Figure S2 shows the regularized parameter set for the synthetic example along with the corresponding clustering. Figure S3 consists of four subfigures, and shows the regularization paths for the complete parameter set in the biological example. The plots are sorted so that the parameters least variable between the 14 cell lines are displayed first. Figure S4 shows the 25 most significant parameter-drug sensitivity pairs.

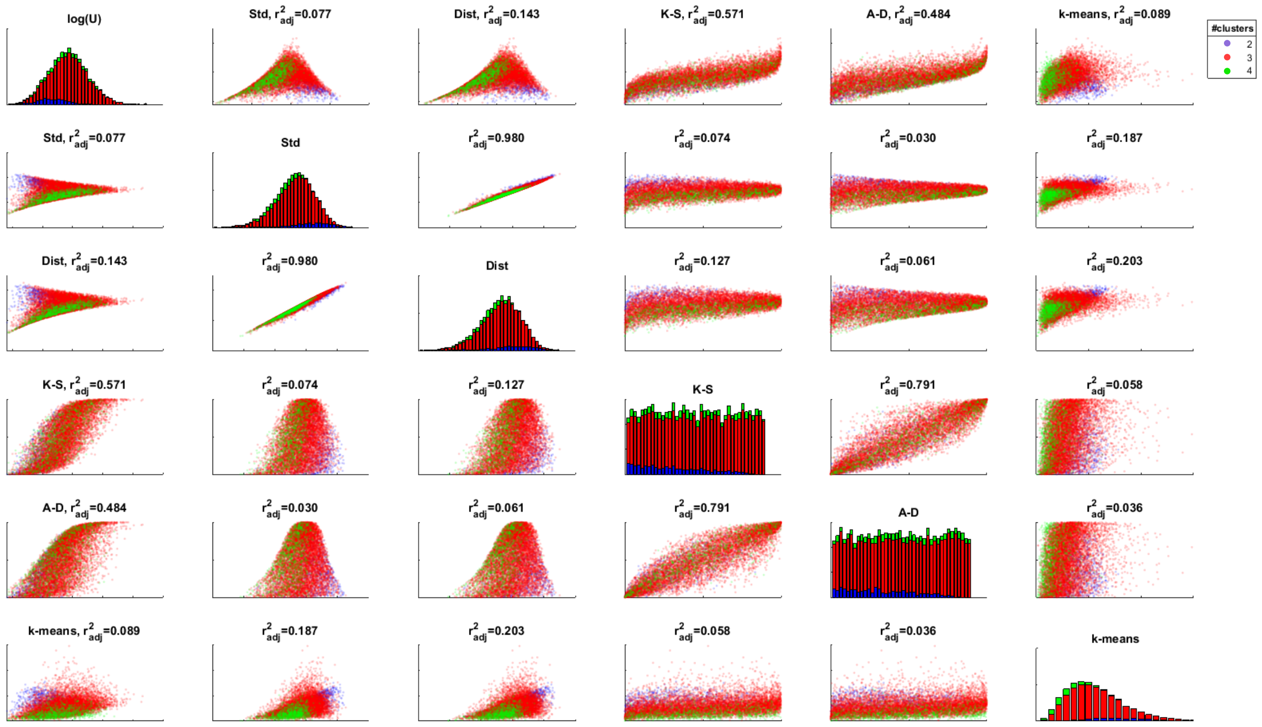

Figure S1: Correlation plot matrix for the different metrics computed for sets of 10 uniformly distributed random values. U: Uniformity. Std: standard deviation. K-S: p-value of the Kolmogonov-Smirnov test. A-D: p-value of the Anderson-Darling test. k-means: sum of the intra-cluster distances to centroid, with the number of clusters determined by the k-means + elbow method. ρ^2_{adj}: adjusted Pearson correlation coefficient.

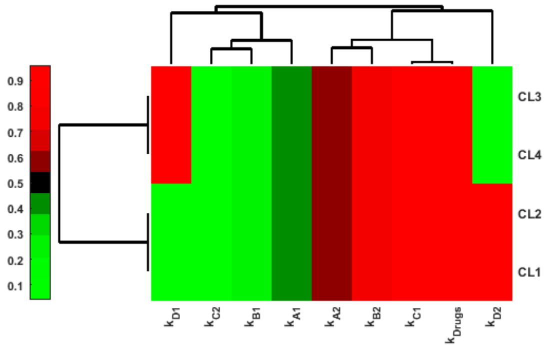

Figure S2: Optimal parameter set for the synthetic dataset example. The dendrogram on the left shows the inferred grouping of the four cell lines two-by-two. The colorbar indicates the parameter values.

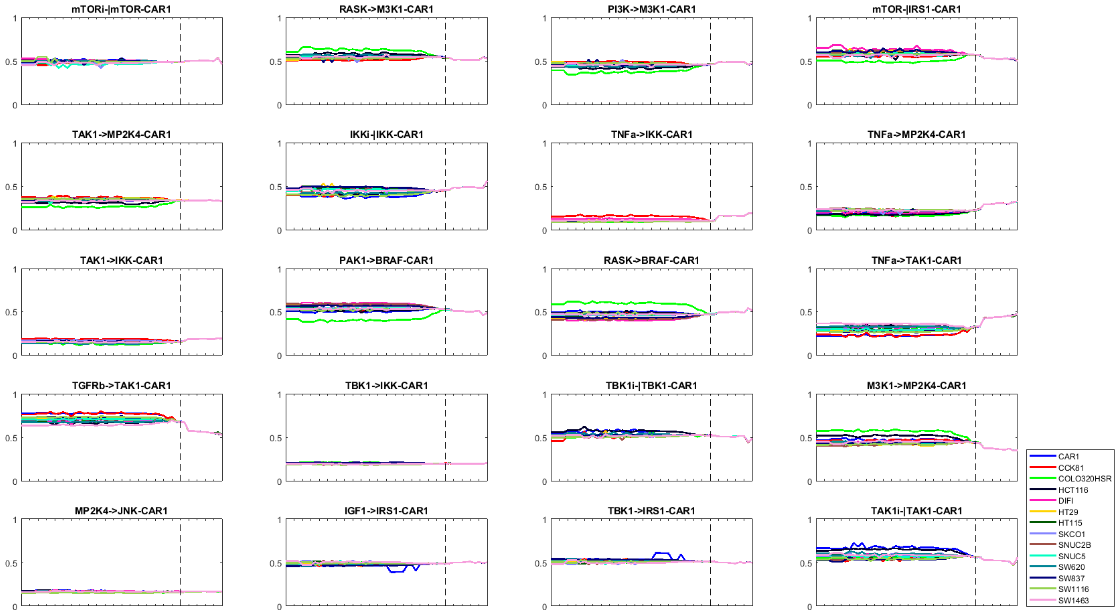

Figure S3: Regularization paths for the 20 least variable parameters of the biological network model. Each plot displays the parameter values for each cell lines over the range of regularization strengths ($2^{-20}$to 2^{5}). The dotted line denotes the optimal model

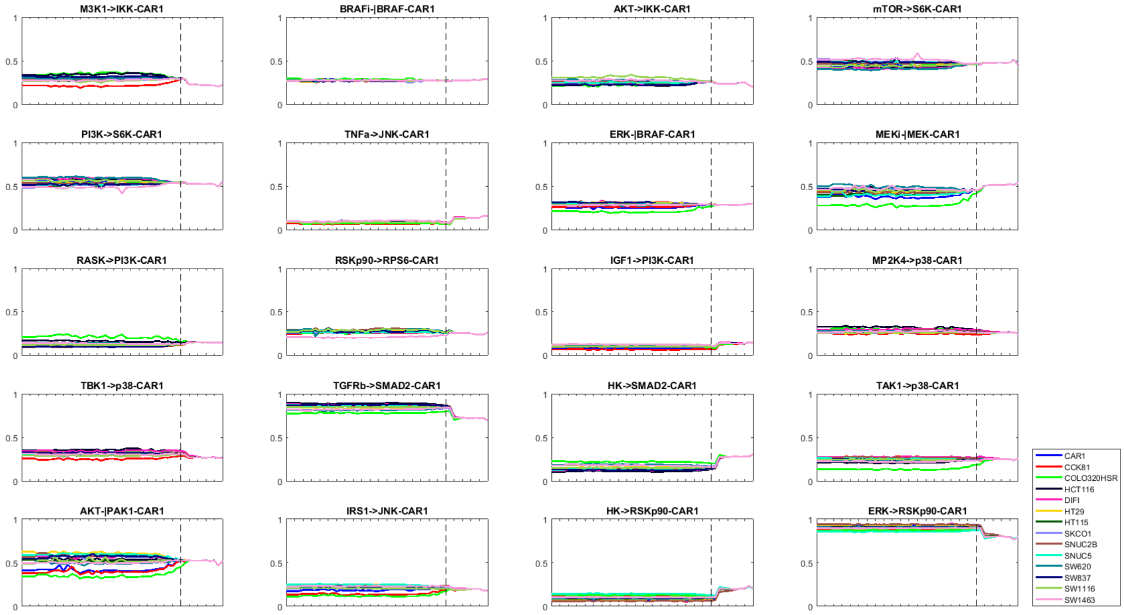

Figure S3 (continued): Regularization paths for the next 20 parameters of the biological network model. Each plot displays the parameter values for each cell lines over the range of regularization strengths (2^{-20} to 2^5). The dotted line denotes the optimal model

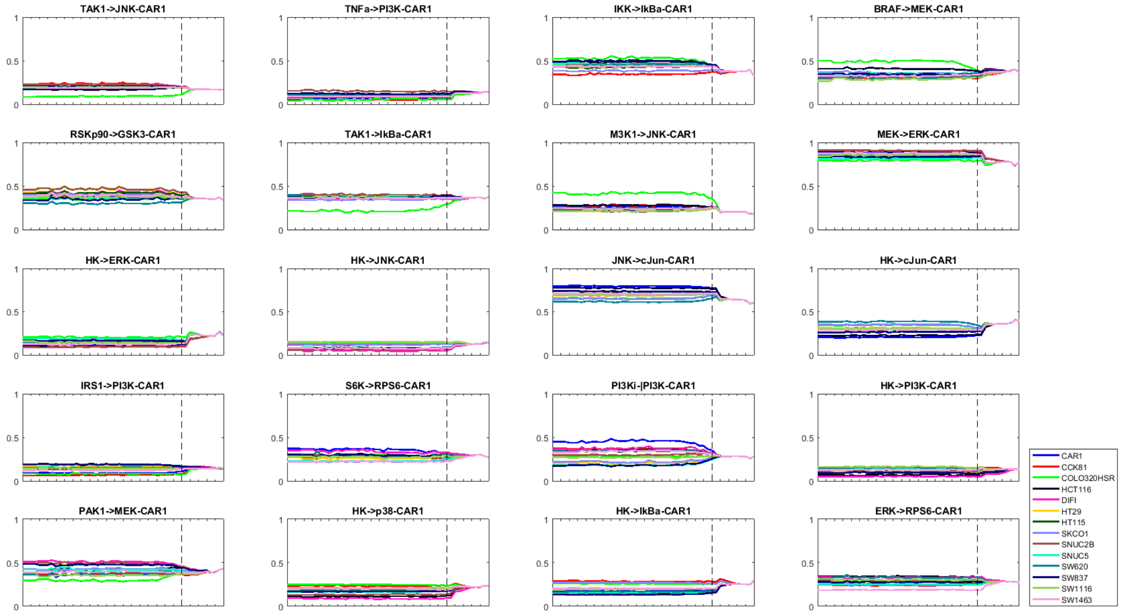

Figure S3 (continued): Regularization paths for the next 20 parameters of the biological network model. Each plot displays the parameter values for each cell lines over the range of regularization strengths (2^{-20} to 2^{5}). The dotted line denotes the optimal model

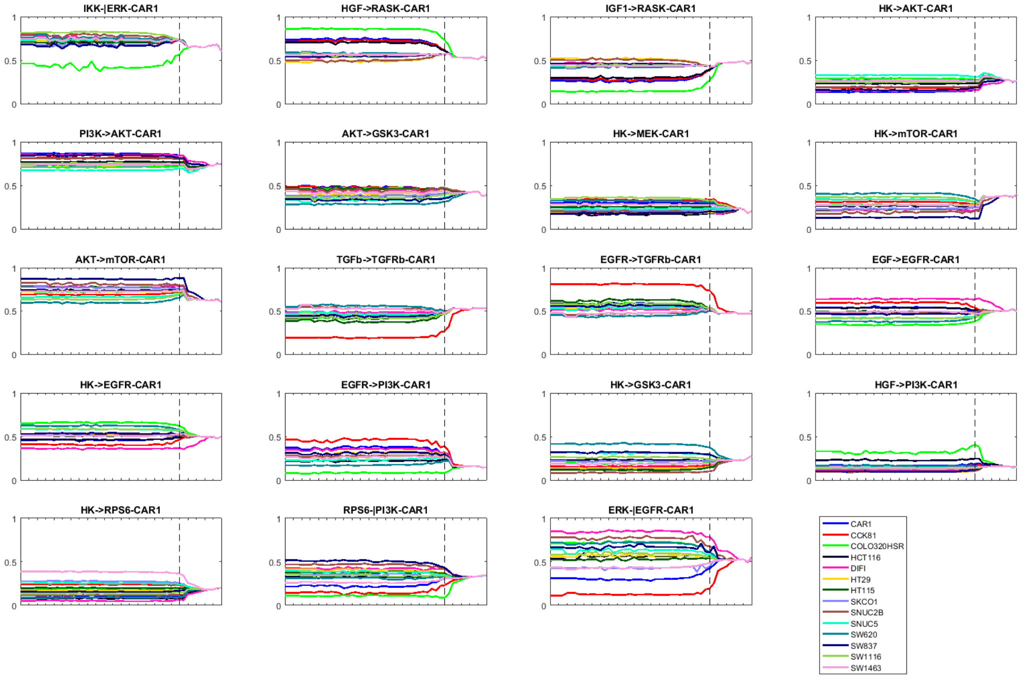

Figure S3 (continued): Regularization paths for the most variable parameters of the biological network model. Each plot displays the parameter values for each cell lines over the range of regularization strengths (2^{-20} to 2^{5}). The dotted line denotes the optimal model

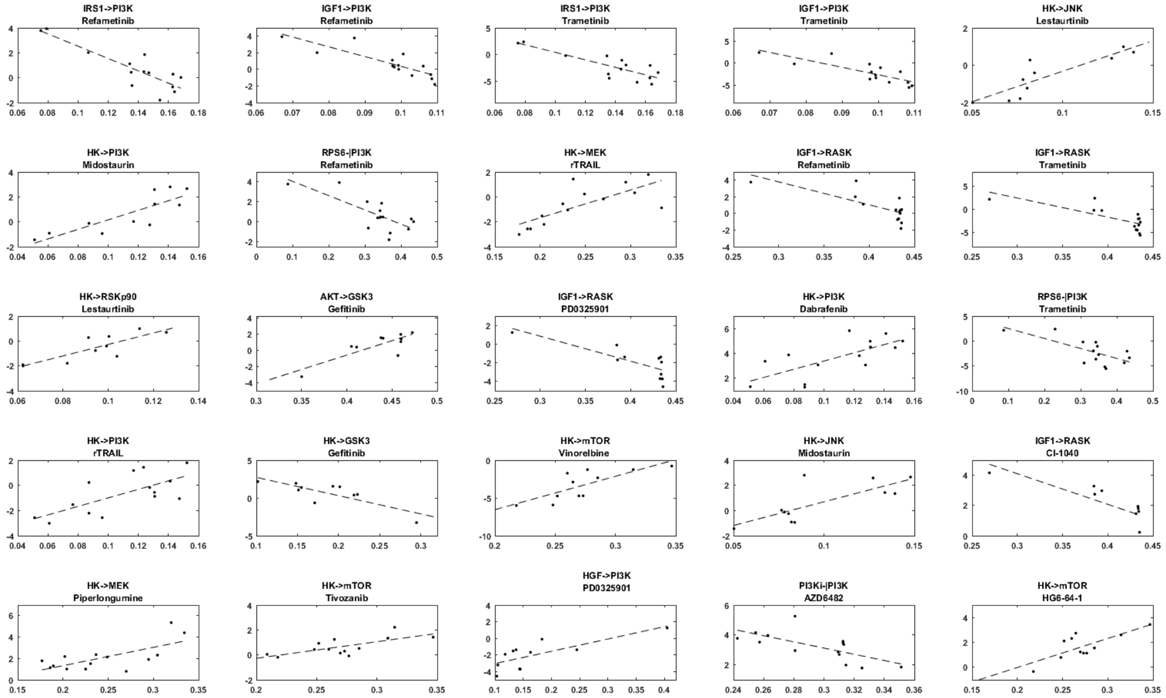

Figure S4: Correlations between model parameters and drug sensitivities across the 14 cell lines. X-axis: parameter value. Y-axis: $\log_e$(IC50). The 25 most significant correlations are shown out of 2573 possible correlations.
